# Strong hole-doping and robust resistance-decrease in proton-irradiated graphene

**DOI:** 10.1038/srep21311

**Published:** 2016-02-18

**Authors:** Chul Lee, Jiho Kim, SangJin Kim, Young Jun Chang, Keun Soo Kim, ByungHee Hong, E. J. Choi

**Affiliations:** 1 Department of Physics, University of Seoul, Seoul 130-743, Republic of Korea; 2 Department of Chemistry, College of Natural Sciences, Seoul National University, Seoul 151-747, Republic of Korea; 3 Department of Physics, Sejong University, Seoul 143-747, Republic of Korea; 4 Soft Innovative Materials Research Center, Korea Institute of Science and Technology, Eunha-ri san 101, Bongdong-eup, Wanju-gun, Jeollabukdo, 565-905, Republic of Korea

## Abstract

Great effort has been devoted in recent years to improve the electrical conductivity of graphene for use in practical applications. Here, we demonstrate the hole carrier density of CVD graphene on a SiO_2_/Si substrate increases by more than one order of magnitude to *n* = 3 × 10^13^ cm^−2^ after irradiation with a high energy 5 MeV proton beam. As a result, the dc-resistance (R) of graphene is reduced significantly by 60%. Only a negligible amount of defect is created by the irradiation. Also the hole-doped low resistance state of graphene remains robust against external perturbations. This carrier doping is achieved without requiring the bias-gate voltage as is the case for other field effect devices. We make two important observations, (i) occurrence of the doping *after* the irradiation is turned off (ii) indispensability of the SiO_2_-layer in the substrate, which leads to a purely electronic mechanism for the doping where electron-hole pair creation and interlayer Coulomb attraction play a major role. A flux-dependent study predicts that an ultrahigh doping may be obtained by longer irradiation. We expect the irradiation doping method could be applied to other atomically thin solids, facilitating the fundamental study and application of the 2d materials.

Graphene holds great promise in the future of electro-optical device applications, thanks to the outstanding transport^1^,^2^,^3^, optical^4^,^5^,^6^, and mechanical properties^7^,^8^,^9^ of free-standing graphene. Unfortunately, a substrate may act as a limiting factor, with, for instance, an increased resistance *R* ~ 1 kΩ/□ for graphene on SiO_2_/Si, whereas much lower values of the order of a few hundred Ω/□ are required for industrial touchscreen^10^ and transparent conductors^11^,^12^,^13^,^14^ applications. Ongoing research thus continuously tries to find methods to reduce the high-dc resistance of this material. One such attempt is to increase, through doping, the low carrier density (*n* = 1 ~ 2 × 10^12^ cm^−2 ^15,16,17.) in charge of the hole-conduction of graphene on SiO_2_/Si. For example, Bae *et al.* investigated chemical doping by using the molecular adsorption of an acidic gas such as HNO_3_ onto graphene^10^. Ni *et al.* synthesized a hybrid device using a ferroelectric polymer and graphene in which the carriers were introduced through a ferroelectric gating mechanism^18^. Strong carrier doping of graphene is important not only for application but also for fundamental physics; for instance, superconductivity can emerge in graphene at ultra-high doping of *n* = 10^14^ cm^−2^ − 10^15^ cm^−2 ^19,20.

In literature, the idea of irradiating solids with ionized particles has led to important breakthroughs. 2 MeV-proton irradiation induced room-temperature magnetic ordering has been observed in bulk graphite crystal^21^,^22^. In 1-d carbon nanotubes, the dc-conductance can be driven from a metallic to insulating regime by Ar^+^ ion irradiation^23^. Also, for ZnO-nanowire devices, the I-V response curve can be tuned by controlled 10 MeV-proton irradiation^24^. In the first case, the irradiation creates defects in the lattice that carry magnetic moments, whereas the physics of the last case are considered to be driven solely by an irradiation induced electrical mechanism.

In this Article, we report on a new route for carrier doping, by utilizing high energy proton (H^+^) particles. We have irradiated the graphene/SiO_2_/Si system with 5 MeV proton beam, which leads to an increase of the hole-carrier density by more than an order of magnitude up to *n* = 3.0 × 10^13^ cm^−2^, and a significant decrease of the dc-resistance. We also find that, despite the high energy H^+^-irradiation, little lattice damage is caused to the graphene crystal structure, suggesting a purely electrical origin for the observed phenomenon. Furthermore, the low resistance remains robust under ambient conditions. To understand the nature of the hole-doping effect, we perform a series of experiments: we measure real-time change of resistance upon turning on, maintaining, and turning off the beam. Also we study whether the doping behavior depends on substrates by employing various materials for the substrate. On the basis of these and other related experiments we will propose mechanism for the irradiation-driven doping as to be described later. We further investigate the relation between the carrier doping and the irradiation time, which suggests that even higher densities could be accomplished by longer irradiation times.

## Results and Discussion

### Transport and optical characterization

**Figure 1:**
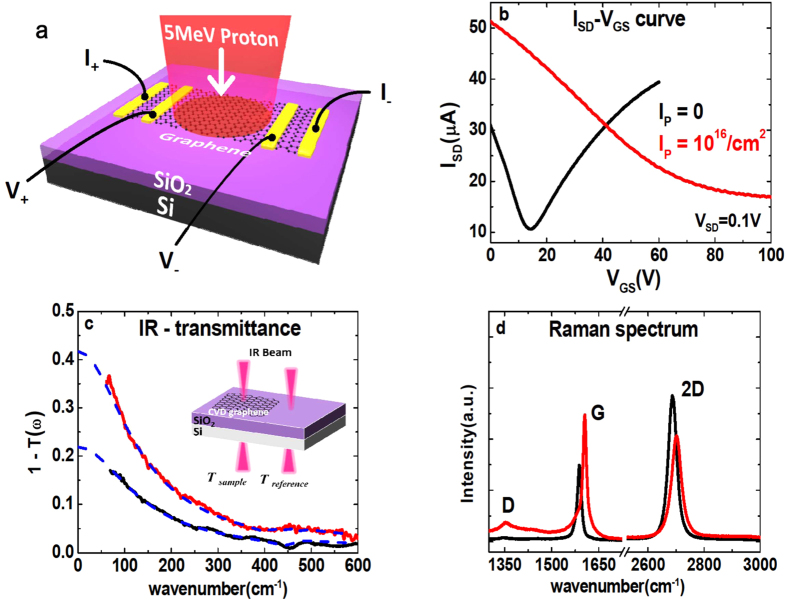
(**a**) Schematic illustration of proton irradiation on mono-layer CVD graphene. The other panels depict the characterization of CVD graphene before and after the irradiation through a I-V transport curve (**b**), a infrared transmission spectrum (**c**) and a Raman phonon spectrum (**d**). In (**c**), the dashed curve corresponds to a fit to the Drude model. The inset shows a schematic representation for measuring the transmission through graphene, (T_*s*_), and the substrate, (T_*r*_), from which T(*ω*) = T_*s*_/T_*r*_ is calculated. In (**d**), the G-peak shifts from *ω* = 1587 cm^−1^ to 1605 cm^−1^ and the 2D-peak shifts from *ω* = 2687 cm^−1^ to 2701 cm^−1^. An irradiation flux I_*p*_ = 10^16^ ions/cm^2^ is used for (**b**–**d**).

Figure 1 presents the transport and optical properties of graphene after irradiation. The I-V curve (b) reveals that the charge neutral point has shifted from *V*_*CNP*_ = 15 V to >100 V, indicating a drastic increase of the hole-carrier density. From the dc-capacitor relation 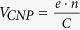 (*C* = 1.1 × 10^−8^ F · cm^−2^ for 300 nm-thick SiO_2_), we deduce the hole density *n*_*i*_ = 1.1 × 10^12^ /cm^2^ and *n*_*f*_ > 7.2 × 10^12^ cm^−2^, where *i* and *f* stand for *before* and *after* irradiation, respectively. To independently confirm the density increase, we measure the optical transmission *T*(*ω*) in the far-infrared frequency range for another irradiated sample. Figure 1(c) displays the Drude absorption 1 − *T*(*ω*) due to free carriers in graphene. The peak is fit using the Drude conductivity 
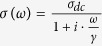
, which divulges that the dc-conductivity *σ*_*dc*_ increases from 1.2 × 10^−3^ (Ω^−1^) to 3.2 × 10^−3^ (Ω^−1^) after irradiation. The width of the Drude peak, *γ*, represents the carrier scattering rate (=1/*τ*, with *τ* the scattering time) which changes from 120 cm^−1^ to 100 cm^−1^. *γ* decreases after the irradiation due to increased *n*, *i.e.*, the scattering is reduced because the carriers screen the charge impurity scattering centers more effectively for higher *n*^25^. The relation 
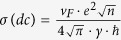
 gives us *n*_*i*_ = 2.9 × 10^12^ cm^−2^ and *n*_*f*_ = 3.0 × 10^13^ cm^−2^. The final state density *n*_*f*_ is ten times larger than the initial density *n*_*i*_. Figure 1(d) shows the Raman phonon spectrum. The G-peak and 2D-peak shift to higher frequencies after irradiation, respectively by Δ*ω* = 18 cm^−1^ and Δ*ω* = 14 cm^−1^. Previous works teach us that the blue shifts of the G and 2D peaks occur when graphene is hole-doped. One can determine the hole density based on the carrier-controlled Raman measurements^26^,^27^, which give for our Raman data that *n*_*i*_ ~ 2 × 10^12^ cm^−2^ and *n*_*f*_ ~ 2 × 10^13^ cm^−2^. The intensity of the two peaks I(G) and I(2D) increases and decreases respectively, a behavior which again roots in hole-doping. Our transport, infrared and Raman phonon measurement consistently indicate that the hole carrier density has increased 10-fold by the H^+^-irradiation, which leads to the low-resistance of CVD graphene. The Raman D-peak amplitude increases slightly in the final state suggesting some defects are created by the irradiation. However, the I(D)/I(G) ratio is only about 0.07, corresponding to a very small defect concentration.

### Real time resistance

**Figure 2:**
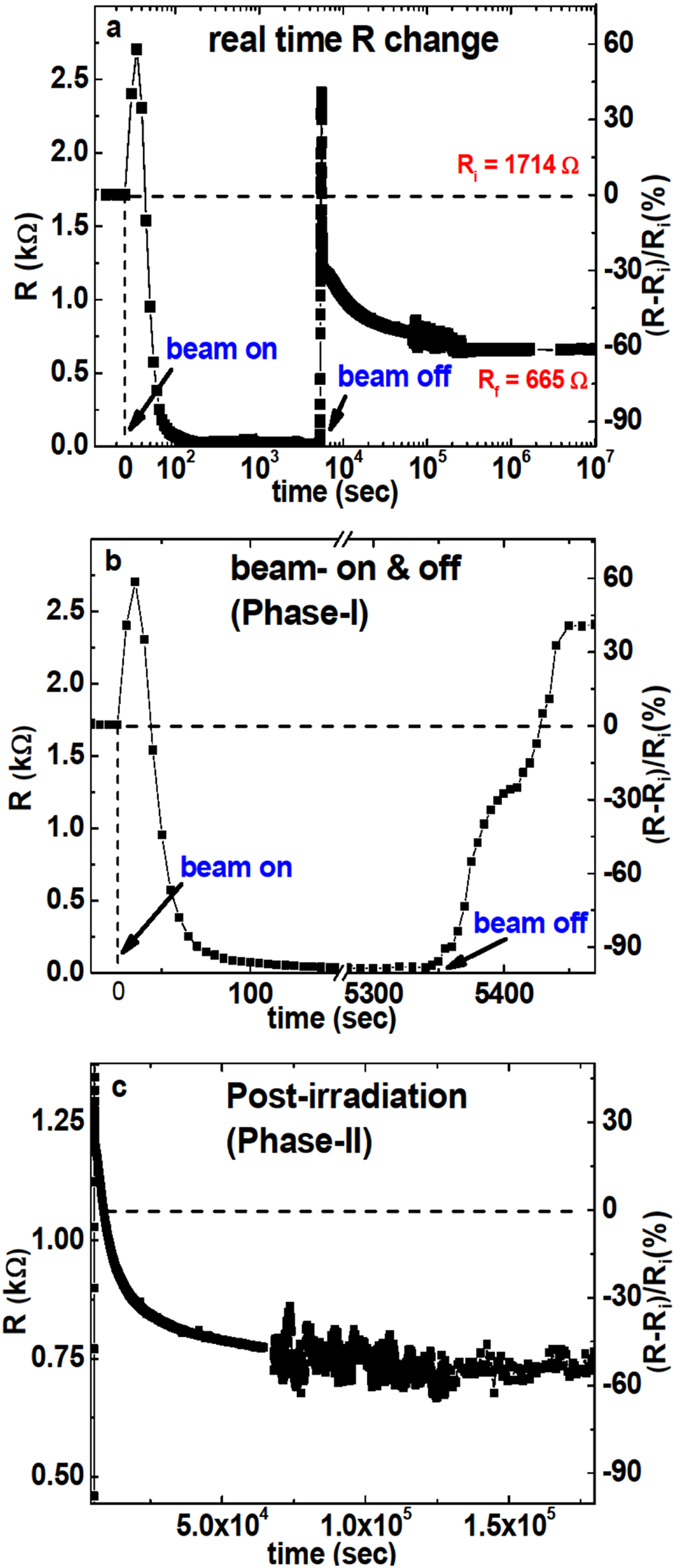
Real-time resistance of graphene measured under the 5 MeV-proton irradiation. (**a**) Overall behavior of R taken over a long time, *i.e.* 100 days. The time dependency is plotted using a logarithmic scale. (**b**) Evolution of R when turning the proton beam on and off. (**c**) R after the irradiation is completed. (**b**,**c**) are defined as the irradiation Phase-I and Phase-II, respectively. For each panel, the relative change R(t)-R_*i*_/R_*t*_ is shown on the right-vertical axis.

To better understand the *R*-decrease, we perform real-time 4-probe resistance measurements during the irradiation. Figure 2(a) shows that when the H^+^-beam is turned on, *R* rises shortly before strongly dropping until saturation at *R* = 30Ω, which is remarkably low compared to the initial resistance *R*_*i*_ = 1.7 *k*Ω. When the beam is turned off, R rises back and then decreases again. This second phase of *R*-decrease occurs spontaneously and stabilizes at *R*_*f*_ = 665Ω. In overall, the resistance was thus reduced by more than 60%, Δ*R*/*R*_*i*_ = −0.61.(Δ*R*≡*R*(*t*) − *R*_*i*_). The strong hole-doping in the final state is taking place during the second phase. The low-R state, which has been monitored for more than one year, remains stable in air without any notable change. We also tested whether this state can be altered by various perturbations. The resistance value remains robust after both annealing the sample at high temperature (*T* = 250 °C) for 1.5 h and exposing the sample to UV radiation. Additionally, we prove that the system is in a charge neutral state by connecting a copper wire between the graphene sheet and the ground, which did not modify the resistance. Figure 2(b) shows that the R-decay during the irradiation phase lasts for ~100 s. In contrast, the spontaneous H^+^-free R-decay occurs over a much longer time, ~40 hours [Fig. 2(c)], implying that the two effects follow fundamentally different mechanisms. For later convenience, we define the beam on/off period and the spontaneous R-decay period as Phase-I and Phase-II, respectively.

### Substrate dependence

**Figure 3:**
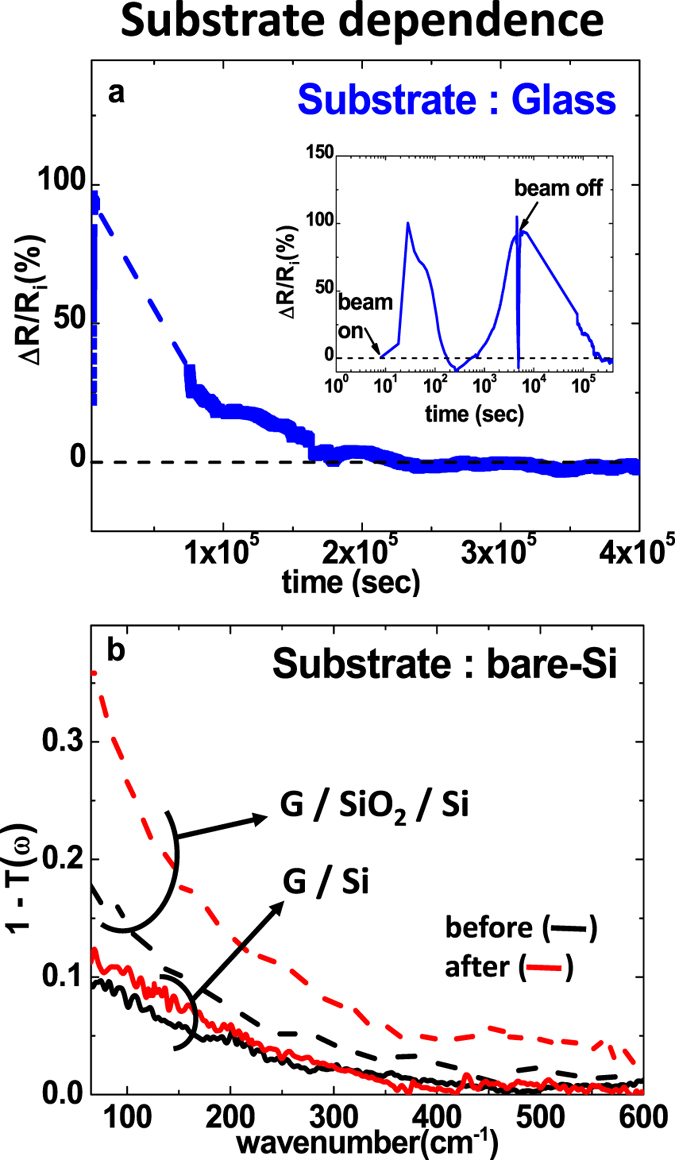
Substrate-dependent study of the irradiation effect. (**a**) real time resistance of graphene on a glass substrate in the Phase-II period. The inset of (**a**) shows an overall change of resistance in real-time. (**b**) far-IR transmission of graphene on a bare Si substrate in comparison with the SiO_2_/Si substrate case.

As part of the efforts to understand the mechanism behind the strong hole-doping, we investigate how the value of *R*_*f*_ depends on the chosen substrate. A series of graphene samples are prepared on various substrates where the irradiation is performed with the same I_*p*_. The result are summarized in Fig. 3 and Table 1. For the glass and the sapphire (Al_2_O_3_) substrate, the low-R state is absent. *i.e.*, R decreases spontaneously in Phase-II, but the final *R*_*f*_ is the same as *R*_*i*_ suggesting that perhaps a low-R behavior may occur for SiO_2_/Si only. SiO_2_/Si substrates are used with different Si-doping type (p-Si and n-Si) and different Si-doping level (low-doping, *ρ* = 10 Ω · cm and high-doping, *ρ* = 0.01 Ω · cm). In these systems, the low-R is reproduced with the same magnitude irrespective of doping type. Finally, to resolve the role of SiO_2_ and Si separately, we transferred graphene on a bare-Si substrate and performed the irradiation. Here R-measurement is not a proper probe because current flows dominantly through the weakly doped Si which obscures precise change of R in graphene. Instead we measure far-IR transmission which detects the Drude change of graphene only. The far-IR transmission shows that the Drude peak for G/Si sample barely changes after the irradiation indicating an absence of hole-doping in contrast with the clear Drude increase for the G/SiO_2_/Si system. These results suggest that SiO_2_/Si is essential to achieve the hole-doped low-R state and that SiO_2_ is playing an important role.

### Possible mechanism

#### Phase-I

High energy protons are known to interact primarily with nuclei of the target solid. The excited nuclei relax energy by creating secondary excitations such as phonons, photons, plasmon, etc^28^,^29^,^30^. In our case, we consider among others the excitation in which valence electron is excited into the conduction band of graphene. As the conduction electron and hole are created the dc-resistance decreases similarly to the photo-conduction effect, which explains the R-decay upon turning on the H^+^-irradiation. When the irradiation is turned off, the electron and hole recombine with each other and R returns to the initial resistance R_*i*_. We develop this idea into a quantitative model and use the theoretical result to explain the real-time R-change in Phase-I which is described in detail in Supplementary-I.

#### Phase-II

**Figure 4:**
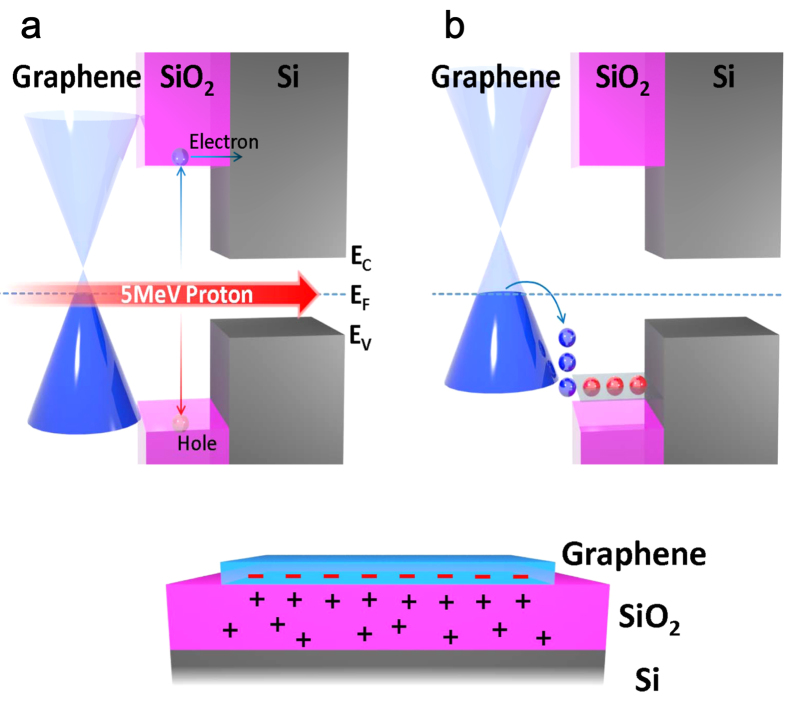
Proposed model for the proton-induced R-decrease in the Phase-II. (**a**) As a secondary process of energy dissipation, electron-hole pairs are created in SiO_2_ layer. The high mobility electrons move quickly to Si. (**b**) Low-mobility hole is trapped inside SiO_2_. The trapped holes attracts and binds with the electrons in graphene. As result the hole carrier density in the Dirac band increases. (**c**) Spatial distribution of trapped hole and electrons in the sample.

The creation of the electron-hole pair due to irradiation occurs not only in graphene but also in the SiO_2_ and Si as well. For graphene and Si they disappear when the beam is turned off through recombination process. However in SiO_2_ the e-h pair follows a different fate due to strongly asymmetric carrier mobilities: The conduction electron in SiO_2_ is highly mobile (*μ*_*e*_ = 20 cm^2^/V · s) and some of them can move to Si^31^ before recombination. In contrast the hole mobility is very poor (*μ*_*h*_ ~ 10^−6^ · *μ*_*e*_) and also has larger effective mass than electron. As a result the uncompensated holes are trapped inside SiO_2_. The hole-trapping and accumulation increase as the irradiation is continued. The trapped hole can diffuse through SiO_2_ by thermal assistance. When it reaches the G/SiO_2_ interface the hole binds with the electron in graphene via Coulomb attraction [see Fig. 4(b)]. Consequently, the Fermi energy in the Dirac band shifts down and the Drude hole density in graphene increases. The hole-diffusion and hole-electron binding occur slowly even after the irradiation is turned off. Therefore R decreases spontaneously over long-time as observed in Phase-II. For graphene on the substrates without SiO_2_-glass, Al_2_O_3_, and Si-the spatially separation of the e–h pair does not take place and consequently the R-decrease effect is absent.

### Expanding the experiment

A few subsidiary but important issues of this experiment need to be clarified.

### Role of the embedded charge of H^+^

**Figure 5:**
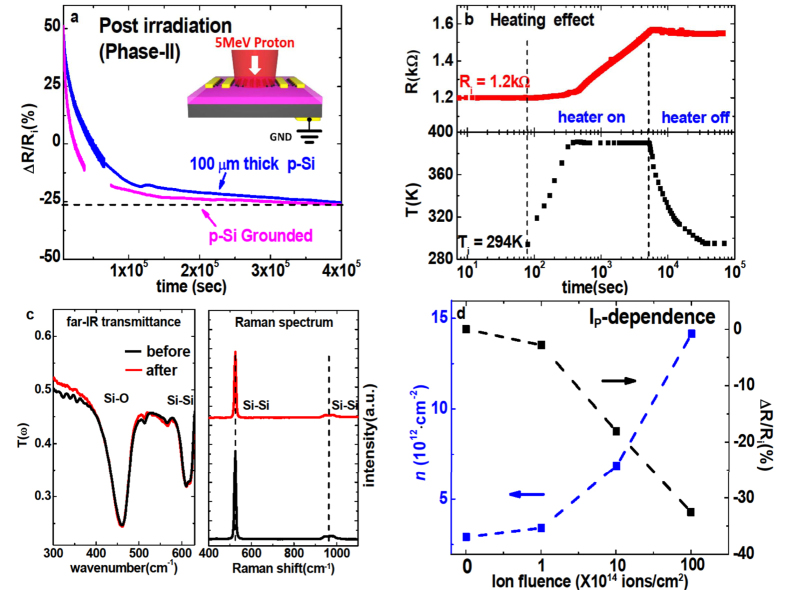
(**a**) R-decrease of the irradiated graphene during Phase-II on a thin substrate SiO_2_/100 *μ*m-Si (blue) and on the electrically grounded substrate SiO_2_/500 *μ*m-Si (magenta, shown by the inset). (**b**) dc-resistance of graphene following the changes in temperature T = 300 K → 390 K → 300 K. No irradiation was performed here. (**c**) Infrared and Raman phonon spectra for the bare substrate SiO_2_/Si before and after irradiation with I_*p*_ = 10^16^ ions/cm^2^. (**d**) Carrier density *n* and relative resistance ΔR/R_*i*_ measured for I_*p*_ = 0, 10^14^, 10^15^, 10^16^ ions/cm^2^. In this experiment we used 6-layered graphene.

A recent theoretical GEANT4 simulation showed that 5 MeV-protons lose their incident energy completely and stop inside Si at a ~200 *μ*m depth from graphene (e.g. Garam Han, 2015, unpublished data). To clarify the role of these embedded protons in the R-change, we transferred graphene on a thinner SiO_2_/100 *μ*m-thick Si substrate for which the H^+^ beam should completely penetrate through the substrate and avoid the existence of embedded protons. We also prepared a G/SiO_2_/500 *μ*m-Si sample where Si is electrically grounded to earth (inset of Fig. 5(a)) so that the charged H^+^ can be neutralized immediately before charge-accumulation. For both cases the resistance measurement is shown in Fig. 5(a). It showed a R-decrease by 27%, in contrast to 61% in Fig. 2 under the same irradiation I_*p*_ = 10^16^ ions/cm^2^, demonstrating that proton accumulation significantly enhances the hole carrier in graphene. A possible explanation of this behavior is that the embedded H^+^ attract the conduction electron in SiO_2_ toward Si, accelerating their separation from the holes before the e–h recombination which leads to an increased hole-trapping in SiO_2_.

#### Heating effect

High flux irradiation may heat up the sample via energy transfer^32^,^33^. To test whether the observed R-change is driven by a possible T-rise, we measured the real-time R evolution while heating the sample in a thermal chamber without giving the irradiation. Figure 5(b) shows that R *increases* by the heating and *decreases* during cool-down. This behavior is opposite to the proton-induced one in Fig. 2, leading to the conclusion that the T-rise can be excluded as origin for the R-change.

#### Defect in the substrate

Proton irradiation creates vacancy, dislocation, and other forms of defect in solid^34^,^35^,^36^. They change the local crystal symmetry which often renders silent phonons to become IR- and/or Raman-active. We measured IR and Raman spectra of the bare SiO_2_/Si substrate before and after irradiation and found no difference [see Fig. 5(c)], indicating that defect density is very small for the considered irradiation flux I_*p*_ = 10^16^ ions/cm^2^.

#### I_*p*_ dependence study

Further hole-doping of graphene could be expected by longer irradiation. We have prepared a series of samples with systematic increase of irradiation I_*p*_ = 0, 10^14^ ions/cm^2^, 10^15^ ions/cm^2^, and 10^16^ ions/cm^2^ and characterized the hole-density by the resistance and far-IR transmission measurements. Figure 5(d) shows that the hole-density *N* increases with a logarithmic I_*p*_-like dependence, instead of the linear-dependence. If we assume that the log I_*p*_-increase is continued at higher I_*p*_, values of *n* = 10^15^/cm^2^ and R = 100 Ω are expected at I_*p*_ ~ 10^21^ ions/cm^2^. Such ultrahigh value of *n* would be of great interest due to possible superconductivity predicted from the band structure and associated van-Hove singularity in graphene^20^. One concern however is that the amount of defects increases rapidly by the proton irradiation for I_*p*_ ≥ 10^17^ ions/cm^2^ according to the previous works^37^, which may deteriorate the transport property of graphene.

## Conclusion

We demonstrated that graphene on SiO_2_/Si substrate becomes strongly hole-doped by irradiation with a 5 MeV proton beam. Electrical transport, infrared transmission, and Raman measurements showed consistently that the hole density increases by 10-times and the dc-resistance is decreased by 60%, for an irradiation flux I_*p*_ = 10^16^ ions/cm^2^. We emphasized that only a very weak amount of defects is created by the irradiation and the hole-doped low resistance remains robust against external perturbations such as moisture, thermal heating, and UV-light. We carried out an extensive investigation on the origin of the irradiation-induced effect, which revealed that (i) the doping occurs over a long time *after* the irradiation is turned off, (ii) the SiO_2_-layer in the substrate is indispensable for the effect to take place. On the basis of these findings, we proposed the electron-hole pair creation and the graphene-SiO_2_ interlayer Coulomb attraction as the underlying mechanism for the irradiation-induced strong hole doping. Also, from a detailed I_*p*_-dependent study, we predicted that a much higher doping density and lower R would be achieved by longer irradiation. This could not performed in this work due to practical limitations of the beam facility. When compared with a conventional field effect gating or an ion-gel electronic double layer (EDL) gating, the current gating-free irradiation method does not require to apply bias voltage to dope carrier. One could perform the proton irradiation on a bare SiO_2_/Si substrate and transfer the graphene later on, which, owing to the slow post-irradiation doping mechanism, should yield the doping without any irradiation-driven defects at all in graphene. Also, by using a micro proton beam confined over a small area, one may create a local doping at a desired location, which might be useful in devising various graphene devices. Traditionally, high energy irradiation has been regarded as a tool of defect- or impurity-related physics and engineering. However, our work demonstrated that it can be used to control the electrical property of a solid as well- graphene in this case-which can be applied to other atomically thin solids such as transition metal dichalcogenides (MoS_2_, WSe_2_, etc) and black phosphorene, offering new opportunity for fundamental and application study of the 2d materials.

## Methods

### Sample fabrication

Large scale monolayer graphene (1 cm × 1 cm) was synthesized by the CVD method under low pressure and high temperature^11^, where a polymethyl methacrylate(PMMA) layer is spin-coated on the as-grown graphene on a Cu-foil. Then, the Cu-layer is etched out using a 0.1 M ammonium persulfate (NH_4_)_2_S_2_O_8_ solution. After rinsing using DI water, graphene was transferred onto the different types of substrate, including SiO_2_/Si and Al_2_O_3_. The detailed transfer techniques are explained in ref. 38,39. To measure the electrical transport of graphene, metal electrodes(Ti 5 nm/Au 100 nm) were deposited and patterned using an electron-beam evaporator and a SUS-301 metal mask, respectively.

### Proton irradiation

A 5 MeV-Proton beam was used for the irradiation under vacuum conditions (10^−3^ torr) in the MC-50 cyclotron at KIRAMS (Korea Institute of Radiological and Medical Sciences). Beam current and spot diameter were set to 170 nA and 3.5 cm, respectively. The maximum irradiation time allowed by such instrumentation is limited to 90 min for safety reasons, which corresponds to a flux I_*p*_ of 10^16^ ions/cm^2^.

### Transport measurement

The I_*SD*_ − V_*GS*_ curve was measured using the electronic equipment *Keithley 2400*. The source-drain channel width and length are both equal to 3 mm, and the applied source-drain voltage was maintained at 0.1 V. The real-time resistance of graphene was measured using the four-point probe method. It was obtained by setting a constant current source of 0.1 mA between the electrodes on both sides and by monitoring the voltage between these electrodes using the same electronic equipment. A 5 mm aperture was used to prevent proton irradiation into the electrode during the real time R measurements. To avoid radioactivity hazard, the measuring equipment was installed in a separate room with long electrical wires connecting the sample.

### Optical measurement

The optical transmission in the Far-infrared frequency region was measured using a commercial Fourier Transform Infrared Spectrometer (FTIR, *Bomem DA8*) and a 4.2 K-cooled bolometric detector. The Raman spectra were recorded with a *WITec CRM200* Raman system using a Nd:YAG laser (532 nm) as excitation source in which the laser power was set to a value below 0.1 mW in order to avoid heating.

## Additional Information

**How to cite this article**: Lee, C. *et al.* Strong hole-doping and robust resistance-decrease in proton-irradiated graphene. *Sci. Rep.* **6**, 21311; doi: 10.1038/srep21311 (2016).

## Supplementary Material

Supplementary Information

## Figures and Tables

**Table 1:** Four probe resistance of the CVD graphene on various substrates.

	substrate		Graphene		
sample number	type	Sid oping-level (Ω · cm)	R_*i*_ (kΩ)	R_*f*_ (kΩ)	ΔR/R (%)
1		*ρ*_*Si*_ = 10	1.71	0.66	−61
2	SiO_2_/p-Si	*ρ*_*Si*_ = 10	1.11	0.34	−69
3		*ρ*_*Si*_ = 0.01	1.69	0.69	−59
4	SiO_2_/n-Si	*ρ*_*Si*_ = 0.01	1.55	0.55	−64.5
5	Glass	—	1280	1260	−1.5
6	Al_2_O_3_	—	470	400	−15

R_*i*_ = initial resistance before irradiation. R_*f*_ = final resistance after irradiation.
