# Supplementary material for: Strong hole-doping and robust resistance-decrease in proton-irradiated graphene
Source: Sci Rep. 2016 Feb 18;6:21311. doi: 10.1038/srep21311 (PMC4758087; doi:10.1038/srep21311)
Supplement: Supplementary Information [file srep21311-s1.pdf]

Supplementary Information for

Strong hole-doping and robust resistance-decrease in proton-irradiated graphene

Chul Lee[1], Jiho Kim[1], SangJin Kim[2], Young Jun Chang[1], Keun Soo Kim[3], ByungHee Hong[2], E. J. Choi[1,*]

[1] *Department of Physics, University of Seoul, Seoul 130-743, Republic of Korea.*

[2] *Department of Chemistry, College of Natural Sciences, Seoul National University, Seoul 151-747, Republic of Korea.*

[3] *Department of Physics, Sejong University, Seoul 143-747, Republic of Korea.*

**Corresponding author*

I. Real time resistance change in Phase-I

The irradiated protons interact with the solid creating various excitations in the nuclei, lattice, and electronic bands[2–5]. In this paper, we consider, among others, the excitation of electron from valence band to conduction band to explain the time-dependent resistance change in Phase-I. The number of the electron-hole pairs Δn induced by irradiation per unit time is proportional to the incident proton flux Φ_p, d(Δn)/dt = $C_1 \cdot \Phi_p$. These pairs are further annihilated through recombination in a rate proportional to Δn. These two effects lead to the time dependent equation for Δn, d(Δn)/dt = $C_1 \cdot \Phi_p$ - $C_2 \cdot (\Delta n)$. The solution to this equation indicates an increase of the carriers during the irradiation ($\Phi_p > 0$) as $\Delta n(t) = \frac{C_1 \cdot \Phi_p}{C_2}(1 - e^{-t/\tau})$ with τ = 1/C_2. When the irradiation is ocer ($\Phi_p = 0$), the carrier density decays by $\Delta n(t) = \frac{1}{C_2} e^{-t/\tau}$, τ = 1/C_2.

This $\Delta n(t)$ leads to time dependent change of dc-conductivity $\Delta\sigma(t)$. If the electron-hole pairs are created in the classical (non-Dirac) bands as sketched in the inset, $\Delta\sigma(t)$ is related with $\Delta n(t)$ as $\Delta\sigma(t)$ = a$\cdot\Delta n$(t) (a=$\frac{1}{m} \cdot \frac{e^2}{\gamma}$ which follows from the Boltzmann relation $\sigma = \frac{n}{m} \cdot \frac{e^2}{\gamma}$. We compare this model prediction with experimental data. The experimental $\Delta\sigma(t)$ is calculated from R(t) by $\sigma(t) \sim$ 1/R(t) and $\Delta\sigma$(t) = σ(t) - σ(0). Fig.S1 shows that the fit (dashed curve) is in reasonable agreement with data: the dc-conductivity increases with time upon the irradiation and it decays when the beam is turned off. However, we find that the time constant for the $\Delta\sigma$-increase and $\Delta\sigma$-decay are distinctly different, τ = 10 ms and τ = 90 ms respectively although they are expected to be the same τ = 1/C_2 as shown above in deriving $\Delta n(t)$. Also, the time constants τ = 10 ms (90 ms) are anomalously long for the e-h pair creation and annihilation considering that they occurs on a much shorter time scale of the order of the nano-second or less as known, for example, in the photo-conduction experiments. We conclude from these obserbvations that our simple model alone is not enough to explain the time-dependent behavior of R(t) and more complicated excitations are taking place during the irradiation. Further study is needed to understand the real time R-change in Phase-I.

If we assume that the e-h pairs are created mostly in the Dirac band, $\Delta\sigma(t)$ follows a different Δn(t)-dependence $\Delta\sigma(t)$ = A$\cdot\sqrt{\Delta n(t)}$ from the relation $\sigma = \frac{v_F \cdot e^2 \sqrt{n}}{4\sqrt{\pi} \cdot \gamma \cdot \hbar}$. We fit $\Delta\sigma(t)$-data using this model but finds that the agreement becomes poor compared with the case of the e-h pairs in the classical band.

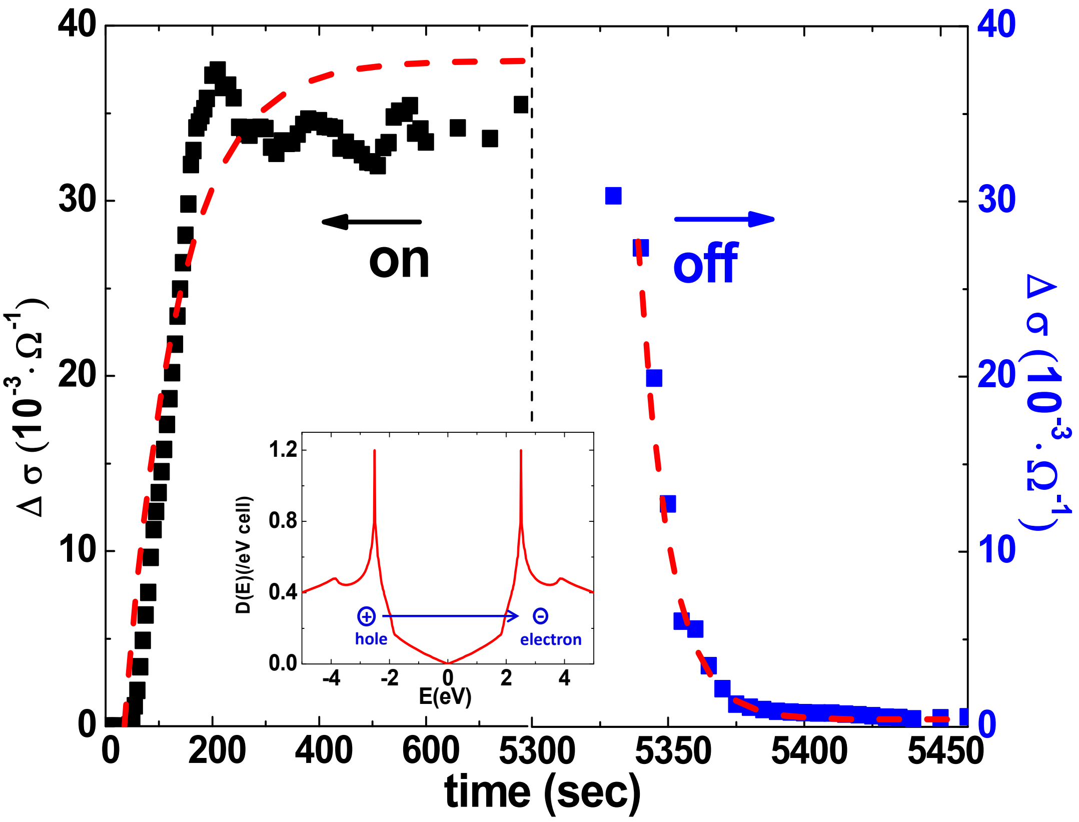

FIG. S1: Real time conductivity change $\Delta\sigma(t)$ in the Phase-I. $\Delta\sigma(t)$ increases when the irradiation is turned on (left figure) and decreases when turned off (right figure). Dashed curves show the fitting result obtained from the simple excitation model. Inset shows schematically the electronic density of state D(E) of graphene[1] and the proton-driven creation of an electron-hole pair.

II. Durability of proton effect in high temperature

To test whether the low-R state remains or lost at high temperature, we measure the R of proton irradiated graphene while increasing temperature from room-T to ∼120C. The result shows that (Fig. S2, red curve) R increases gradually as T is raised. From T = 290 K to T = 120 °C (390 K), R(T) increased from R = 620 Ω to R = ∼750 Ω). The low-resistance has increased by ∼17.8 % in term of relative change. For comparison we measured R(T) of pristine graphene (no irradiation) which shows similar behavior (blue curve). Here the initial R at room-T is high, R = 1100 Ω (because it is not irradiated by proton) and it increases by similar amount 19.7 % at T= 120 °C. This result shows directly that low-R of proton irradiated graphene remains robust at high-T.

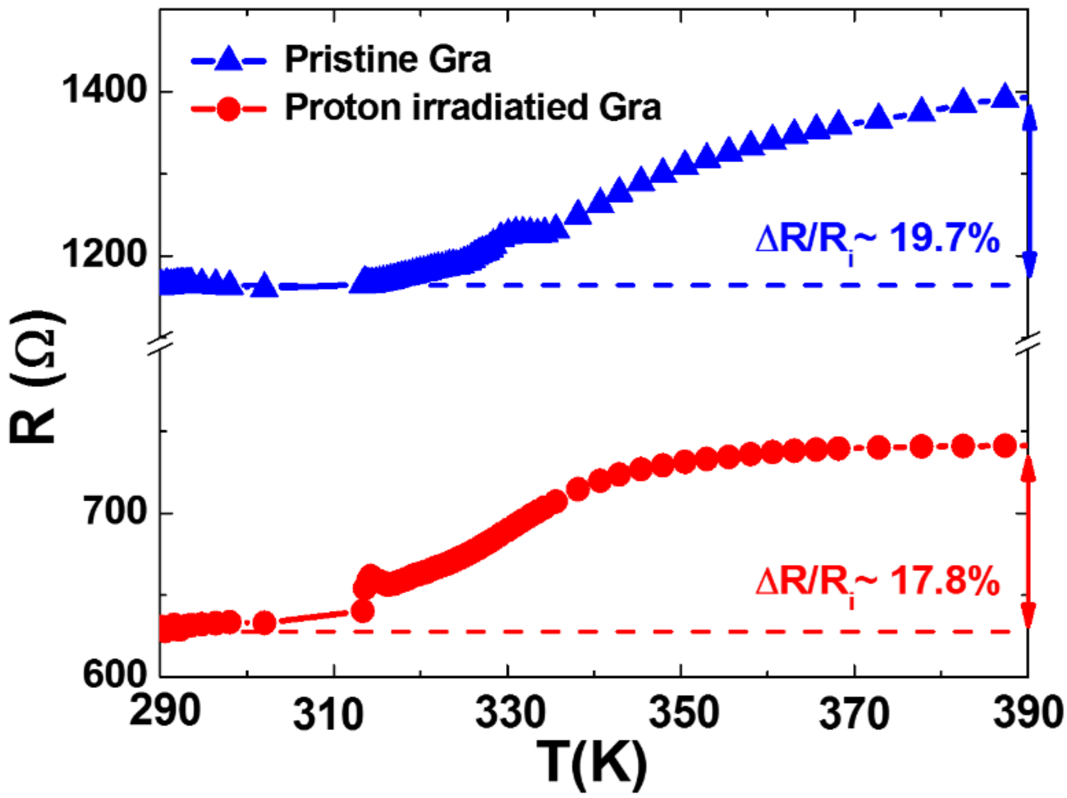

FIG. S2: Resistance of the pristine (blue) and proton irradiated (red) graphene as function of Temperature from RT to 390 K.

Reference

1. Hobson, J. P. & Nierenberg, W. A. The Statistics of a Two-Dimensional, Hexagonal Net. *Phys. Rev.* **89**, 662 (1953).

2. Miotello, Antonio., Kelly, Roger. & Dapor, Maurizio. Beam Interactions with Materials and Atoms. *Nucl. Instr. and Meth. B* **141**, 16-24 (1998).

3. Goldhaber, A. S. & Heckman, H H. High energy interactions of nuclei. *Annu. Rev. Nucl. Part. Sci.* **28**, 161-205 (1978).

4. Schwank, J. R. *et al.* Radiation Effects in MOS Oxides. *IEEE Trans. Nucl. Sci.* **55**, 18331853 (2008).

5. Schardt, Dieter. & Elssser, Thilo. Heavy-ion tumor therapy: Physical and radiobiological benefits. *Rev. Mod. Phys.* **82**, 383-423 (2010).
